# *Escherichia coli* Removal in Biochar-Modified Biofilters: Effects of Biofilm

**DOI:** 10.1371/journal.pone.0167489

**Published:** 2016-12-01

**Authors:** A. R. M. Nabiul Afrooz, Alexandria B. Boehm

**Affiliations:** 1 Department of Civil and Environmental Engineering, Stanford University, Stanford, California, United States of America; 2 Engineering Research Center (ERC) for Re-inventing the Nation's Urban Water Infrastructure (ReNUWIt), United States of America; University of Notre Dame, UNITED STATES

## Abstract

The presence of microbial contaminants in urban stormwater is a significant concern for public health; however, their removal by traditional stormwater biofilters has been reported as inconsistent and inadequate. Recent work has explored the use of biochar to improve performance of stormwater biofilters under simplified conditions that do not consider potential effects of biofilm development on filter media. The present study investigates the role of biofilm on microbial contaminant removal performance of stormwater biofilters. *Pseudomonas aeruginosa* biofilms were formed in laboratory-scale sand and biochar-modified sand packed columns, which were then challenged with *Escherichia coli* laden synthetic stormwater containing natural organic matter. Results suggests that the presence of biofilm influences the removal of *E*. *coli*. However, the nature of the influence depends on the specific surface area and the relative hydrophobicity of filter media. The distribution of attached bacteria within the columns indicates that removal by filter media varies along the length of the column: the inlet was the primary removal zone regardless of experimental conditions. Findings from this research inform the design of field-scale biofilters for better and consistent performance in removing microbial contaminants from urban stormwater.

## Introduction

Low-impact development (LID) includes natural treatment systems designed to capture, treat, and recycle urban stormwater to restore the urban hydrologic regime to the pre-urbanization stage. Stormwater biofilters are a common type of LID. They can remove contaminants from stormwater via porous media filtration and other processes and discharge the filtrate to the underlying groundwater aquifer or via a drain to surface waters. Traditionally, biofilter media consists of a native block of soil or compost mixed with sand to reduce head loss through the biofilters [1, 2]. Removal of fecal indicator bacteria (FIB), common stormwater pollutants, in conventional biofilters is reported to be inadequate and inconsistent [3].

A number of recent studies [4–6] has explored the use of biochar—a pyrogenic carbon compound produced via anoxic pyrolysis of waste biomass—as an alternative biofilter medium. Biochar is attractive for this purpose because it has high porosity and specific surface area yet low cost [7]. Biochar is known [7, 8] to be an efficient adsorbent for removing common inorganic and organic contaminants from water through hydrophobic action, physical filtration, and electrostatic interaction. The superior efficiency of biochar-augmented sand biofilters over sand biofilters in removing FIB from stormwater has been also tested recently [8–10]. However, none of these studies has considered the effect of biofilm development on the FIB removal performance of biochar-modified biofilters. Therefore, results from previous studies may have limited transferability in predicting removal efficiency of biochar-modified biofilters under field conditions where biofilm is likely to be present.

Exposure of any filter-media to natural stormwater is likely to encourage formation of biofilm on the collector grains and within their pore spaces. Formation of biofilm, in addition to introducing collector surface heterogeneity, may influence the hydrodynamics of the flow through the porous media [11]. The presence of extracellular polymeric substances (EPS) on collector grains is known to alter roughness, hydrophobicity, and electrokinetic properties of the collector surface [12–14]. Such changes in physicochemical properties of the porous media due to biofilm formation will influence surface interaction between suspended particulates and the filter media, thereby changing contaminant removal efficiency of filter media [15–19].

The effect of biofilm formation on the transport of micron- and submicron- sized particles has been described previously in the colloid literature. The role of biofilm in the transport of bio-particles has also been studied in various porous media, including sand [20, 21], activated carbon [22], and glass beads [23, 24]. However, a consensus or complete mechanistic understanding regarding the influence of biofilm on bacterial mobility in porous media is yet to be achieved. For example, while biofilm formation on glass beads has been reported to facilitate *Escherichia coli* adhesion [24], there are studies [25, 26] that report otherwise and present hindered *E*. *coli* adhesion to silica or glass surfaces in the presence of lipopolysaccharide (LPS).

The growing body of literature describing microbial transport in biofilm-coated porous media suggests the following factors potentially determine the role of biofilm in microbial transport [13, 19, 20, 23, 25, 27–30]: porous media type, the microbial community present within the biofilm, availability of nutrients, and biofilm age. While the presence of biofilm facilitated microbial transport in glass bead-packed rapid filters, higher microbial removal was observed in sand-packed columns in the presence of biofilm [19, 24]. In addition, enhanced attachment of bacterial cells was reported for biofilm with higher thickness compared to relatively thinner biofilm [23]. Other factors, including a rougher collector surface, better availability of nutrient and existence of more diverse microbial community (i.e., extracellular polymeric substances with hydrophilic as well as hydrophobic functional groups) have been also reported to increase bacterial removal in the presence of biofilm [27, 29, 30]. These studies, however, have been conducted primarily in simplified systems using de-ionized (DI), well, or wastewater as the solution chemistry for microbial suspension [20, 23, 24] and sand-packed columns. The understanding of the effect of biofilm on microbial filtration in porous media will be advanced by doing experiments using diverse solution chemistries and filter media.

This is the first study that investigates how biofilm presence affects the efficacy of biochar-augmented biofilters for removing *E*. *coli* from stormwater. Biofilters were subjected to laboratory-grown biofilm forming bacteria under nutrient-rich conditions for 120 h to simulate potential biofilm formation in stormwater biofilters. In this study, the effect of filter media type (sand vs. biochar) on biofilm formation and the role of biofilm on the removal efficiency of the biofilters were evaluated. Moreover, we also considered the effects of natural organic matter (NOM) on FIB removal. Further, biochar and sand particles were characterized for their physicochemical properties (i.e., hydrophobicity, surface roughness, specific surface area) and Derjaguin, Landau, Verwey, Overbeek (DLVO) modeling was performed to estimate the electrostatic interaction between the collectors and suspended *E*. *coli* cells. Thus, in addition to investigating how biochar-augmented biofilters may operate when biofilm is present, this study provides fundamental insights into the mechanisms (e.g., electrostatic interaction and hydrophobic attachment) whereby biofilms affect microbial transport through porous media.

## Experimental Methods

### Preparation of the porous media

A 7:3 mixture of sand and biochar (by volume) was used as porous media for the laboratory scale biofilters. This volumetric ratio was selected based on the current recommended proportion (20–40%) for organic matter in biofilter soil media [31]. Coarse Ottawa sand (20–30 mesh, Fisher Scientific) was soaked for 4 h in de-ionized (DI) water and pre-cleaned by sonication-rinsing 10 times to remove all suspended colloids. Each of these cycles lasted for 7 minutes (5 minutes sonication+ 2 minutes rinsing). The cleaned sand was then soaked overnight in concentrated hydrochloric acid (HCl) to remove any metallic impurities. The sand was then washed with DI to adjust the pH to 6.9±0.2. Next, the cleaned sand was saturated in 100 mM CaCl_2_ and washed with three times to further ensure removal of pre-existing colloids in the sand. Cleaned sand was then dried (85°C, overnight), autoclaved (121°C, 100 KPa, 15 min), and stored in a sterile container.

Biochar particles were procured (from Swallow Valley Farm, Valley Ford, CA), crushed, sieved (to separate the #30 mesh (595 μm) or larger fraction), and autoclaved (121°C, 100 KPa, 15 min) for sterilization. Biochar particles with a particle size less than the mesh size were used as media amendment for the biofilters. Fine biochar particles were retained for use in the columns as a previous study showed they were important in removal of *E*. *coli* from stormwater [8] According to the manufacturer, the feedstock for the biochar was a blended mix of wood species consisting of 60% Monterey Pine, 20% Eucalyptus, 10% Bay Laurel, 10% mixed hardwood and softwood. Pyrolysis was performed over a 6 h period at a temperature ranging from 180 to 395°C.

### Characterization of porous media

The filter media (both sand and biochar particles) were characterized for their hydrophobicity, surface roughness, and surface area. Surface hydrophobicity of the sand and biochar particles were compared in terms of their water contact angles (WCA) measured using a Rame-hart Model 290 contact angle goniometer (Succasunna, New Jersey). The measurement was performed using the DROPImage software instantly after dropping 10 μL of DI water over a layer of filter media glued onto a glass slide. A similar gluing procedure was used to prepare a layer of filter media to determine its surface roughness using a Dektak 150 Surface Profiler (Fremont, California,) using three different levels of stylus force describe by the instrument as 1, 3, and 8 milligrams. Average roughness height was measured taking 40 scans over a length of 1 micron: three such measurements were done for each particle type at each stylus force. In addition, the specific surface area (m^2^/g) of sand and biochar particles was measured using an autosorb iQ (Quantochrome, Boynton Beach, Florida) surface area analyzer using N_2_ physisorption isotherm: this surface area can be termed as Brunauer–Emmett–Teller (BET) surface area. Before absorbing N_2_ onto the filter media, sand and biochar particles were outgassed at 200°C for 9 h and 27 h, respectively, to remove all the moisture.

### Synthetic stormwater

Synthetic stormwater was prepared in the laboratory using a previously reported composition that provides a realistic concentration of all major ions in stormwater runoff [8]: CaCl_2_ (0.11 g/L), MgCl_2_ (0.015 g/L), NaHCO_3_ (0.084 g/L), Na_2_HPO_4_ (0.002 g/L), NaNO_3_ (0.006 g/L), Na_2_SO_4_ (0.047 g/L), and NH_4_Cl (0.004 g/L). For preparing stormwater with NOM, a standard surrogate for NOM, Suwannee River Humic Acid (International Humic Substances Society, MN, USA) was added to this composition at a concentration of 15 mg/L. This provides an average total organic carbon (TOC) concentration of 7.5 mg/L. The prepared stormwater was autoclaved for 15 minutes for sterilization. The pH of the synthetic stormwater was then adjusted to 7.3±0.3 using 0.1 M HCl or 0.1 M NaOH. The NOM concentration and pH used in this study are within the range what we typically observe in urban stormwater runoff [32].

### Preparation of bacterial suspension

A kanamycin resistant strain of *E*. *coli* (NCM 4236) was used as a FIB surrogate. Bacterial strains, stored at -80°C in 25% glycerol, were transferred into 10 mL of Luria broth (LB) and incubated at 37°C for 18 h. 1 mL of the incubated bacteria suspension, cultured to a stationary phase, was separated from the growth media by centrifugation (5000xg for 10 min), suspended in the synthetic stormwater, and stored at 4°C for 17–18 h for acclimatization. The concentration of *E*. *coli* in stormwater ranged from 1.5–5.3 x 10^5^ CFU/mL (without NOM) and from 1.1–1.53 x 10^5^ CFU/mL (with NOM)

### Model biofiltration units

Glass chromatography columns (2.5 cm diameter and 10 cm length, Kontes Glass) with built-in mesh equipped Teflon fittings were used to contain the porous media. The end meshes prevented the media from washing out during stormwater injection. Columns were dry-packed layer-by-layer, where 1-cm layers were poured in and compacted with a wooden rod. Columns were tapped by hand after each layer was poured to reduce stratification (or short-circuiting) in the packed media.

### Formation of biofilm

*Pseudomonas aeruginosa* (strain PAO1-LAC, ATCC 47085) was used as a model biofilm-growing microorganism for the experiments following the work of others [24, 33]. Biofilm was developed in the porous media following a previously established protocol [24] with some modification. Stock bacteria were transferred into 10 mL LB broth and incubated at 37°C for 20 h to reach the stationary phase. 1 mL of bacterial suspension was centrifuged at 5000xg for 10 minutes, separated from the LB broth by 2x rinsing with 1 mM NaCl, re-suspended in 250 mL of fresh LB broth, and then again incubated for 20 h at 37°C.

*P*. *aeruginosa* suspended in a LB broth was injected into the column using a peristaltic pump (Masterflex, L/S, Cole-Parmar, IL) for 12 h followed by 108 h of nutrient solution (defined below) injection at a flow rate of 0.75 (± 0.05) mL/ min. In order to promote the uniform presence of biofilm along the length of the column, the direction of *P*. *aeruginosa* suspended in LB broth and the direction of nutrient solution flow was switched between upflow and downflow every 6 h and 12 h, respectively, as described by Liu et al. 2007 [24] The composition of the nutrient solution has been published elsewhere [33]: CaCl_2_ × 2H_2_O (0.04 g/L), CuSO_4_ ×5H_2_O (0.001 g/L), FeSO_4_ × 7H_2_O (0.001 g/L), KH_2_PO_4_ (2.08 g/L), MgSO_4_ × 7H_2_O (0.5 g/L), MnCl_2_ × 4H_2_O (0.00004 g/L), Na_2_HPO_4_ (2.56 g/L), NH_4_Cl (1 g/L), ZnSO_4_ × H_2_O (0.001 g/L), and L-Threonine (0.18 g/L). Next, at least 10 pore volume (PV) [170–190 mL] of sterile artificial stormwater (without NOM) was flushed through the column to promote removal of loose biomass and residual nutrient solution.

### Biofilm characterization

Biofilm was formed within 2 or 4 columns (depending on experiment) using the abovementioned protocol. At the end of the biofilm formation process, the presence of the biofilm on the porous media was confirmed by sacrificing one (if started with two) or two (if started with four) columns. The remaining columns were used for experiments as described below. The biofilm characterization was performed using an ATP-based biomass quantification method described elsewhere [34]. Briefly, columns were dissected to collect media from the top 4 cm (near outlet), middle 2 cm, and bottom 4 cm (near the inlet) of the column. Each segment of media was gently rinsed using 20 mL of 1 mM NaCl solution. The media were then mixed with a sterile glass rod and 200 mg of media was transferred into a microcentrifuge tube. 500 μL of sterile 1 mM NaCl was then added to the tube, and the mixture was vortexd for 15 s, and incubated at 38°C for 3 minutes. After incubation, 50 μL of pre-incubated (38°C for 3 minutes) Bac-Titer-Glo reagent (Promega Scientific, Sunnyvale, CA) was added to the mixture, and was incubated for another 20 s before relative luminescence was measured using a GloMax Fluorometer (Promega Scientific, Sunnyvale, USA). Each measurement was replicated to ensure reproducibility. The measured RLUs were converted to ATP using an ATP standard curve.

The ATP standard curves were obtained using a previously reported protocol with some modification[34]. Briefly, column media were autoclaved, rinsed in 1 mM NaCl, and weighed in 200 mg fractions. A known concentration of ATP solution (500 μL) was added to the collectors followed by an additional 50 μL of Bac-Titer-Glo reagent. The luminescence of this mixture was measured and documented. Similar measurements were conducted for a series of known ATP concentrations to establish the standard ATP curve.

While the measurement of ATP provides information regarding the amount of biomass deposited within the biofilters, it does not offer any insight on the geometry (i.e., the biofilm thickness, the extent of coating on the collector surfaces).

### Quantification of attached *E*. *coli*

At the end of each column experiment, 2 g of media was collected from the top, middle, and bottom section of a column and placed in a centrifuge tube with 10 mL of 1mM NaCl. The tube was then vortexed for 30 s, shaken vigorously for 10 s by hand, and again vortexed for another 30 s to dislodge[35] the bacterial cells from the surfaces of the filter media. Bacteria counts of the resulting suspension were performed using the spread plate technique (see below).

### Column experiments

Laboratory experiments (S1 Fig) were conducted using sand and biochar-modified sand-packed columns. Details of the columns tested are presented in Table 1. Column experiments were performed in three steps: 1) conditioning of the column with sterile stormwater; 2) feeding 4 PV of *E*. *coli*-contaminated stormwater followed by 4 PV of sterile stormwater (deposition of *E*. *coli*); and 3) injecting 4 PV of DI water (to document the potential release of *E*. *coli* deposited into the secondary energy minima [36]). A Masterflex L/S precision variable-speed peristaltic pump was used for injection of stormwater and DI water. The approach velocity of the water was maintained at 12.3 cm h^-1^ (equivalent to a volumetric flow rate of 1 mL min^-1^). Note that duration of *E*. *coli* -laden stormwater injection was less than 2 h and the influent concentration did not show any significant change within this time period. The porosity of the packed column was determined gravimetrically prior to injecting 4 PV of stormwater. A similar procedure was followed for columns with or without biofilm. All experiments were conducted at room temperature.

**Table 1:** Details of the filtration columns tested.

Experiment	Media	NOM	Biofilm	No. of Columns
A	Sand	No	No	2
B	Sand	No	Yes	2
C	Sand	Yes	No	2
D	Sand	Yes	Yes	2
E	Sand-Biochar	No	No	3
F	Sand-Biochar	No	Yes	3
G	Sand-Biochar	Yes	No	3
H	Sand-Biochar	Yes	Yes	3

Column effluent was collected in 0.33 PV (columns without biofilm) or 0.5 PV (columns with biofilm) fractions using a CF-2 fraction collector (Spectrum Chromatography, Houston, TX). *E*. *coli* concentrations were measured using the spread plate technique with LB agar plates (with 25μg/mL kanamycin). As *P*. *aerugionosa* and *E*. *coli* are both present in some samples, *E*. *coli* were distinguished from *P*. *aerugionosa* visually by their larger colony size. Method experiments confirmed that the presence of *P*. *aerugionosa* at relevant concentrations did not inhibit *E*. *coli* colony growth at relevant concentrations (see S2 Fig).

The detection limit of the spread plate method was 10 *E*. *coli* colony forming units (CFU)/mL thus we could measure ~4 log removal in the columns. Each sample was characterized using 2 decimal dilutions (except for samples with low CFU count, e.g., collected during 0–1, 7–9, and 11–12 PV) with duplicate plates per dilution. Bacterial counts on duplicate plates ranging between 10 to 200 CFU were averaged and reported as observed *E*. *coli* concentrations. Concentration of *E*. *coli* in the effluent obtained during the conditioning of the column was found to be below the detection limit (10 CFU/mL).

Observed effluent *E*. *coli* concentration (C) was normalized by influent *E*. *coli* concentration (C_0_) and plotted against PV to construct a breakthrough curve. Each breakthrough curve was replicated, and the average breakthrough curve was plotted using average C/C_0_ values determined from the replicate columns. Log removal of *E*. *coli* in a column was calculated by performing numerical integration of the average breakthrough curve for a given experimental condition (filter media type—sand or biochar-augmented sand, presence or absence of NOM, and presence or absence of biofilm). All the reported values, including log removal and maximum C/C_0_ (average of the C/C_0_ values observed at the plateau or 2.5–4 PV of a breakthrough curve) values are reported as average ± standard deviation.

The effect of biofilm presence, biochar, or NOM on *E*. *coli* removal was determined by comparing columns conducted in the presence and absence of the treatment. Two sample t-tests were performed with SAS (SAS Institute Inc, Cary, NC) to test if the treatment had a statistically significant effect (p<0.05).

## Results

### Physicochemical properties of the filter media

Table 2 compares the hydrophobicity, roughness, and the surface area of the biochar and the sand. The surface roughness comparison between the media was inconclusive because the measurement could not be compared at the same levels of stylus force [37]. While we needed a high level of stylus force to observe valid roughness measurements for sand particles, biochar particles were not able to withstand the same high levels of force. The biochar particles demonstrated a much higher hydrophobicity (water contact angle, WCA, in °) and specific surface area (m^2^/g) compared to sand particles (Table 2). The sand surfaces were completely wettable indicating a strongly hydrophilic surface. On the other hand, WCA for biochar particles was 106.5±1.5°. The specific surface area of biochar particles (104.64±7.80 m^2^/g) was nearly three orders of magnitude higher than sand particles (0.20±0.04 m^2^/g). It is probable that the entire biochar surface area is not relevant for microbial removal as BET surface area associated with micropores is not accessible to *E*. *coli* for attachment.

**Table 2:** Physicochemical properties of the collectors.

Properties	Biochar			Sand	
Water Contact Angle(°)	106.5±1.5			Completely wetting	
Roughness(nm)	Stylus Force(mg)	^1^R_a_	^1^R_q_	R_a_	R_q_
	1	41.4±22.6	75.5±41.6	0*	0*
	3	92.4±71.9	111.1±81.9	0*	0*
	8	0*	0*	3510.8±365.8	3706.6±391.4
Specific Surface Area(m^2^/g)	104.64±7.80			0.20±0.04	

*indicates indeterminable data either because of too low (sand) or too high (biochar) stylus force.

^1^ R_a_ and R_q_ stand for arithmetic and geometric average roughness, respectively

### Presence of biofilm in the biofilters

**Fig 1:**
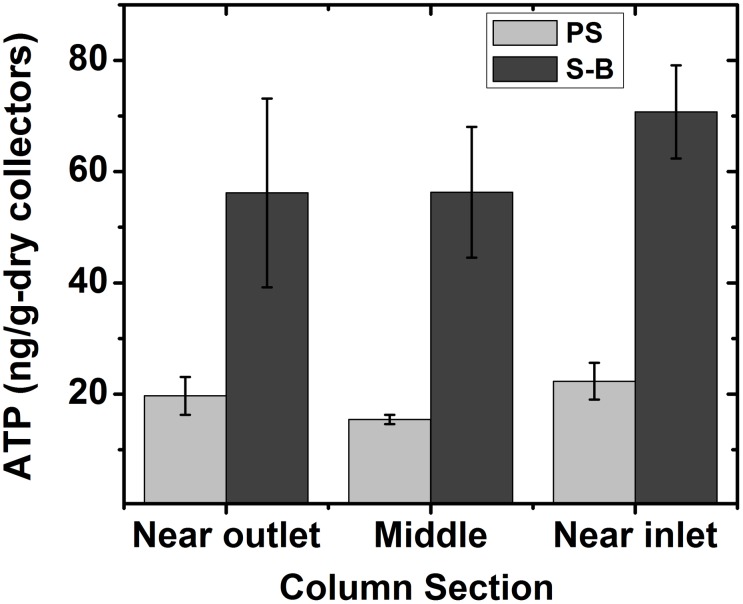
Variation of biomass supported by sand (PS) and biochar-augmented sand (S-B) columns after 120 h. Adenosine triphosphate (ATP) was used as a measure of active biomass present within the porous media. Error bars represents one standard deviation between replicate measurements (n = 3).

The presence of biomass in the sacrificed columns was quantified using ATP density (ng/g-dry collectors). The biofilm was fairly uniform along the length of the column for both types of media (Fig 1). However, supported biomass after 120 h of biofilm maturation was higher (p = 0.001) in biochar-modified sand columns than in sand columns. In sand columns, the density of ATP ranged between 15.4±0.8 to 22.3±3.3 ng/g-dry collectors, while in biochar-modified columns, ATP density ranged from 56.2±16.9 to 70.7±8.4 ng/g-dry collectors. Interestingly, the biochar-amended sand demonstrated increased retention of *P*. *aeruginosa* during the inoculation of column: effluent concentrations from biochar-augmented columns at the end of the *P*. *aeruginosa* injection were one order of magnitude lower: (51±13) x 10^4^ CFU/mL in biochar columns vs. (17±5) x 10^5^ CFU/mL. In addition, at the end of the column experiments, biofilm characterization was also performed for the columns that were not sacrificed. Injecting *E*. *coli* laden stormwater to the biofilm-coated biochar-amended sand columns did not increase observed ATP densities in the columns (S3 Fig), suggesting that the contribution of ATP from *E*. *coli* retention is insignificant compared to the ATP from the *P*. *aeruginosa* biofilm.

### Transport of *E*. *coli* in sand columns

**Fig 2:**
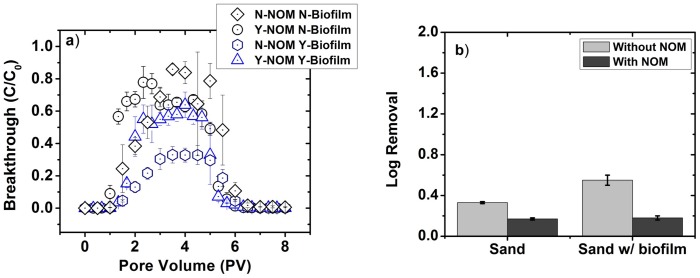
Removal of *E*. *coli* in sand packed 10 cm laboratory columns. Removal is presented as a) breakthrough curves; b) log removal. Log removal was calculated by numerical integration of the breakthrough curve normalized by total pore volume of injected *E*. *coli* laden synthetic stormwater. All experiments were conducted at room temperature. Error bars represent standard deviation between replicate experiments (n = 2). Y- and N- prefix indicate presence and absence (of NOM or biofilm), respectively. For example, N-NOM Y-Biofilm means with biofilm cases in the absence of NOM.

The effect of biofilm on *E*. *coli* removal in a sand biofilter depends on whether NOM is present in the stormwater (Fig 2). In the absence of NOM, *E*. *coli* log removal increases significantly (p = 0.028) from 0.33±0.01 without biofilm to 0.55±0.05 with biofilm.

However, in the presence of NOM, there is no significant difference in *E*. *coli* removal in columns with and without biofilm: observed p-values comparing the average log removal and maximum C/C_0_ are found to be 0.333 and 0.10, respectively. In the presence of NOM, the maximum C/Co was 0.76±0.08 in the presence of biofilm and 0.67±0.06 in the absence of biofilm; average log removal was 0.18±0.02 and 0.17±0.01 in the presence and absence of biofilm, respectively.

### Transport of *E*. *coli* in biochar modified sand biofilters

**Fig 3:**
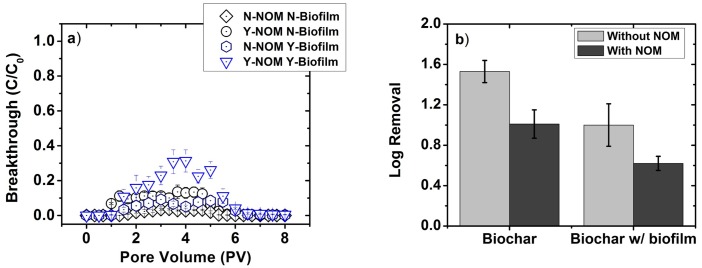
Removal of *E*. *coli* in sand-biochar (70% sand, 30% biochar: by volume) packed 10 cm laboratory columns. Removal are shown as a) breakthrough curves; b) log removal. Log removal was calculated by numerical integration of the breakthrough curve normalized by total pore volume of *E*. *coli* laden synthetic stormwater injected. All experiments were conducted at room temperature. Error bars represent standard deviation between replicate measurements (n = 3). Y- and N- prefix indicate presence and absence (of NOM or biofilm), respectively. For example, N-NOM N-Biofilm means without biofilm cases in the absence of NOM.

The presence of biofilm in a biochar-modified sand biofilters facilitates *E*. *coli* transport through the biofilter. Reduced removal of *E*. *coli* occurs in the presence of biofilm when NOM is both present and absent in the stormwater (Fig 3). In the absence of NOM, maximum C/C_0_ is significantly higher (p = 0.001) in the presence of biofilm (0.07±0.01) than the absence of biofilm (0.03±0.003), and *E*. *coli* log removal is significantly lower (p = 0.035) when biofilm is present (1.01±0.14) compared to when it is absent (1.53±0.11). In the presence of NOM, the maximum C/C_0_ value is significantly higher (p = 0.001) in the presence of biofilm (0.26±0.03) than in the absence of biofilm (0.12±0.01), and the log removal is significantly lower (p = 0.021) in the presence (0.62±0.09) versus absence of biofilm (1.00±0.21).

While presence of biofilm significantly reduces *E*. *coli* log removal in sand-biochar media, note that biochar-modified sand biofilters demonstrate increased *E*. *coli* removal (Fig 3b) compared to sand (Fig 2b) under all experimental conditions.

### Release of *E*. *coli* from the biofilters during DI flush

At the end of full breakthrough (4 PV of *E*. *coli* contaminated water followed by 4 PV of sterile stormwater), each column was flushed with 4 PV of DI and the release of deposited bacteria was monitored. *E*. *coli* release concentrations are plotted against DI pore volume in S4 Fig. Total release percentage was calculated using numerical integration of the release breakthrough followed by normalizing by the total deposited bacteria. For all experimental conditions, the percentage release of bacteria was <10% of the total deposited bacteria.

### Distribution of attached *E*. *coli* in biochar-modified sand biofilters

**Fig 4:**
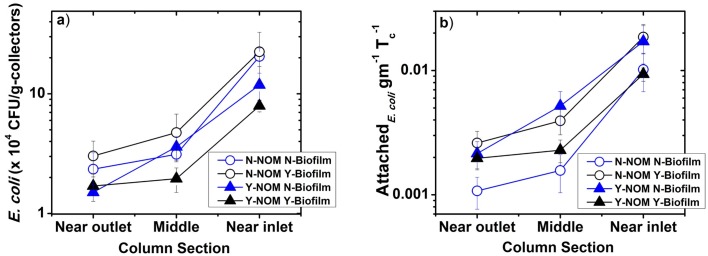
Distribution of retained E. coli bacteria in biochar augmented sand columns. a) *E*. *coli* count per gram of collectors; b) total number influent *E*. *coli* (T_c_) normalized count of bacteria per gram of collectors. Error bars represent one standard deviation between replicate measurements (n = 3). Y- and N- prefix indicate presence and absence (of NOM or biofilm), respectively. For example, N-NOM N-Biofilm means without biofilm cases in the absence of NOM.

Fig 4 presents profiles of the deposited *E*. *coli* within the biochar modified sand columns. Fig 4a shows the number of CFU per g of dry collectors (number density), which are normalized by total CFU applied to the column by the influent stormwater and presented in Fig 4b. The number density of retained bacteria increases with distance from the column inlet (Fig 4). The maximum number density are found attached onto the collectors near the inlet. For example, in the presence of both NOM and biofilm (Y-NOM Y-Biofilm), the bacterial density ranges from (1.69 ±0.43) x 10^4^ CFU/g of dry collectors near the outlet to (7.94 ±0.87) x 10^4^ CFU/g of dry collectors near the inlet of the columns.

## Discussion

### Biochar-amendment increases the biofilm biomass in biofilters

The addition of biochar to sand increased the amount of supported biofilm as inferred from ATP densities in the model biofilters. The greater biomass in the biochar-modified sand columns can be attributed to the unique physicochemical properties of biochar. It has higher specific surface area, surface roughness and hydrophobicity compared to sand. Physicochemical surface properties have been reported to influence the growth of biofilm on solid substrates in a large number of studies [29, 38, 39]. In addition, biochar efficiently retains nutrients [40, 41] which may allow for biofilm proliferation.

### Biochar-amendment increases the *E*. *coli* removal in the biofilters irrespective of the presence of biofilm

Augmenting sand with biochar increased *E*. *coli* removal in biofilters under all experimental conditions. Although this difference decreases in the presence of biofilm, the average *E*. *coli* log removal is still higher in biochar-augmented biofilters than in sand biofilters. While our results corroborate previous studies [8, 10, 42] reporting superior performance of biochar-augmented biofilters over sand biofilters in removing *E*. *coli* from synthetic stormwater, the removal observed in this study is lower than those reported previously: 2.32 ± 0.32 (previous study [10]) vs. 1.53±0.11 (this study in biochar-augmented biofilters without biofilm). The difference in composition of feedstock and pyrolysis temperature during biochar production could be responsible for the observed differences. Increased *E*. *coli* removal in biochar-modified biofilters relative to sand biofilters can be attributed to the higher hydrophobicity and increased available surface area on biochar surfaces compared to sand [11]. Note that, the physico-chemical properties of biochar (i.e., surface area, particle size, hydrophobicity, polarity, etc) vary greatly depending on the biomass source and biochar production method. Therefore, characterization of biochar for their physico-chemical properties followed by bench-scale performance testing for their FIB removal capacity is crucial to choosing biochar for removing FIB from stormwater.

### The presence of NOM in synthetic stormwater reduces *E*. *coli* removal by sand and biochar-amended sand biofilters

The presence of NOM in the influent stormwater reduced *E*. *coli* removal in the columns under all experimental conditions. Our results corroborated previously published studies [43, 44] describing NOM-facilitated bacteria transport through saturated porous media.

NOM can absorb onto a wide range of collectors under saturated conditions due to the presence of both hydrophilic and hydrophobic functional groups in their structure (i.e., carboxylic, phenolic, carbonyl, and hydroxyl) [45]. Absorbed NOM may facilitate bacterial transport in two ways [17, 46]: a) it competes with bacteria for a limited number of available surface attachment sites; and b) it provides a physical barrier to bacterial deposition via steric interaction between NOM-coated collectors and NOM-coated bacteria. These steric interactions in the presence of NOM have been reported [17] to be more important than hydrophobic and DLVO interactions under some conditions. Transport of *E*. *coli* may be enhanced by the steric interactions in both un-coated and biofilm-coated collectors.

### Effects of biofilm on *E*. *coli* removal depends on the type of the filter media and the presence of NOM in stormwater

The presence of biofilm altered *E*. *coli* log-removal in stormwater biofilters. However, the specific nature of the influence depended on the type of media. In sand biofilters, the presence of biofilm either increased (without NOM) removal performance or had no effect (with NOM). On the other hand, in biochar-modified sand biofilters, biofilm reduced removal performance regardless of NOM presence. Changes in *E*. *coli* log removal in a column with a biofilm relative to a column without biofilm can be attributed to one or more of the following factors [13, 27, 28, 39, 47, 48]: a) modified surface properties of the packing media, b) altered hydraulic properties of the packed bed, c) increased number of surface sites, or d) additional modes of adhesion such as polymer-mediated (i.e., steric, hydrogen-bond or dipole-dipole) interactions.

### Multiple mechanisms may contribute to an altered *E*. *coli* deposition in the biofilm-coated biochar-augmented sand

The presence of biofilm on the collector surface is likely to change the collector’s surface properties (i.e., surface roughness, hydrophobicity, and surface charge), which in turn appears to promote (for sand) or inhibit (for biochar-augments sand) *E*. *coli* deposition onto the collector surfaces. It has been reported [27, 48] that bacteria cells are more likely to attach onto a relatively rough surface compared to a smooth surface primarily because of the microscale depressions on a rough surface, which act as convenient deposition sites for bacteria Therefore, the reduced removal capacity of biochar-augmented sand columns with biofilm compared to biochar-augmented sand columns without biofilm would be consistent with the column containing biofilm containing smoother surfaces [39].

Note that reduced *E*. *coli* retention in biochar columns with biofilm may also result from reduced hydrophobic interaction between cells and biofilm-coated surfaces compared to cells and bare surfaces. To test this hypothesis, we may compare the measured hydrophobicity of the sand and biochar particles with the literature-reported hydrophobicity of *P*. *aeruginosa* biofilm. Hydrophobicity of *P*. *aeruginosa* biofilm surfaces—expressed by water contact angle (WCA) ranging from 26-48° [30, 49]—appears to be lower than the hydrophobicity of bare biochar surfaces (> 100°), but significantly higher than completely wettable (~0°) sand surfaces. This may explain why the presence of biofilm increases *E*. *coli* log removal in sand columns, but decreases log removal in biochar modified columns. In addition, the extracellular polymeric substances (EPS) in biofilms are known to possess smaller negative [47] surface potential than bare silica surfaces. This might result in a more favorable DLVO interaction between sand surfaces and the *E*. *coli* cells, thereby increasing deposition of *E*. *coli* onto a biofilm-coated sand surface compared to a bare sand surface.

The presence of biofilm within a column may change column hydraulics by changing pore spaces (both intra- and inter- collector) and number of available surface sites for *E*. *coli* deposition [13, 28] Therefore, higher *E*. *coli* log removal in biofilm-coated sand columns may be attributed to an increase in available surface sites because of the presence of biofilm within the pore spaces. Biofilm-coated sand columns maybe also experience reduced porosity [28] with a corresponding increase in the tortuosity of the stormwater flow through the columns. Given that the specific surface area of biochar particles is several orders of magnitude higher than that of sand particles, the relative increase in surface sites in sand-biochar columns due to biofilm formation can be assumed negligible compared to the same in sand columns. It should be noted that biochar particles may have small pores of which the surfaces are inaccessible to bacteria. Therefore, deposition of a large amount of *P*. *aeruginosa* biofilm onto some of the accessible surfaces may further reduce available surface area for *E*. *coli* deposition.

### Deposition associated with secondary energy minima plays minor role in *E*. *coli* removal using biochar-augmented sand biofilters

Negative charge on the filter media surfaces (both for sand and biochar particles) presents a repulsive energy barrier between *E*. *coli* and collector surfaces. Using literature reported range of negative surface charge for sand and wood-derived biochar particles, 20–30 mV and 20–50 mV respectively [50–52], the range of these barriers was determined following adapted Wiese and Healy expression for a sphere–flat plate system [53, 54] (see S1 Method). Associated electric double layers (relative to thermal energy, KT where K is the Boltzmann constant and T is temperature) can be estimated as 379–661 KT and 217–1070 KT for sand and biochar surfaces (S5 Fig), respectively. Attractive Van-der-Waals forces could lower the resultant DLVO energy barrier to a value as low as 247 and 85 KT for sand and biochar, respectively; however, this is still strong enough for deposition of *E*. *coli* cells onto the primary minima to be unlikely. Despite reports [25, 46] of more accurate soft-particle interaction theory for bio-particles [55] a number of porous media filtration studies [30, 54, 56] described deposition onto secondary minima resulted from DLVO interaction as one of the primary *E*. *coli* removal mechanisms in columns packed with negatively charged quartz sand. However, occurrence of such deposition is primarily described at higher ionic strength condition (>10 mM) compared to the composition of stormwater for this study.

In our study, to explore the role of the secondary energy minima in *E*. *coli* removal from synthetic stormwater, at the end of each experiment, the column was flushed with a 4 PV of DI water which has a lower ionic strength than stormwater: ~0 mM for DI vs. 4.7 mM for stormwater. Introduction of a reduced background ionic strength (i.e., DI) is reported to cause detachment of any bacteria cells deposited into the secondary energy minima [56]. However, a small percent of (1–5%total deposited) released bacteria due to DI flush in our study indicate that imply that number of bacteria removed from the stormwater due to electrostatic interactions with the filter media was small. However, some deposition onto the primary minima may occur because of surface charge heterogeneity [57] of the collector grains and due to wedging effects [58]

## Conclusions

Biofilms are ubiquitous in natural or engineered water treatment systems and may dictate the removal of contaminants during porous media filtration. This study compares the effect of biofilm on the *E*. *coli* removal in non-amended sand and biochar-amended sand biofilters. Results presented in this study suggests the following:

- The effect of biofilm on the stormwater biofilters performance in removing *E*. *coli* depends on the type of filter media. Formation of biofilm may enhance or impair the performance of biofilters designed to improve bacteriological quality of urban stormwater.
- Formation of biofilm may enhance hydrophobic interaction in the hydrophilic collectors (i.e., sandy aquifer with low organic content), it is likely to suppress bacterial removal via hydrophobic interaction if collectors have high organic matter.
- The presence of high level of natural organic matter (in the range of 15 mg/L) in stormwater (i.e., runoff resulting from first flush) will facilitate transport of *E*. *coli* through the sub-surface.
- Conceptual modeling of DLVO forces and insignificant (<10%) release of attached bacteria during DI flush indicate that the secondary energy minima has a minor role on deposition of *E*. *coli* in sand or biochar-amended sand biofilters, at least in case of *E*. *coli* laden simulated stormwater with an ionic strength of 4.7 mM
- The biochar amendment improves the performance of stormwater biofilters with sand media even in the presence of biofilm. However, the benefit of using biochar amendment decreases when biofilm is present.

It should be noted that the effects of biofilm reported in this study are based on biofilms formed by a specific strain of bacteria (*P*. *aeruginosa* PAO1) on sand and biochar particles. While the observed mechanistic role of biofilm on *E*. *coli* removal is supported by other studies in the literature, specific observations in the current study regarding the extent of *E*. *coli* removal may not represent all microbial transport conditions, i.e., biofilms formed by other bacteria, on surfaces with different physicochemical properties, or removal of other FIB. Therefore, research addressing transport of other FIB like *Enterococcus* or pathogens like *Salmonella*, *Cryptosporidium*, and rotavirus in biochar-augmented biofilters are needed under field conditions.

## Supporting Information

S1 Fig Schematic of the column setup. Column experiments were performed at the room-temperature using upflow configuration.
(TIF)

S2 Fig Comparison of *E*. *coli* count on the LB Agar plates in the presence and the absence of *P*. *aeruginosa*. The figure compares the E. coli colony counts between the plates that were spread by *E*. *coli*-*P*.*aeruginosa* mixture and the plates where a pure *E*. *coli* culture were spread. Expected values indicate *E*. *coli* concentrations (CFU/plate) observed when plated in the absence of *P*. *aeruginosa*. Number of colonies observed in the mixed culture plates are shown as *E*. *coli* found (CFU/plate). Error bar represents 1 standard deviation between duplicates (n = 2).
(TIF)

S3 Fig Comparison of total biomass in column sections obtained from biochar-augmented sand columns after injecting *E*. *coli* laden synthetic stormwater. Significantly (p<0.05) higher ATP density (ng/g-dry collectors) was observed in biofilm-coated columns compared to columns without biofilm Error bars represent standard deviation between replicate measurements (n = 3). Y- and N- prefix indicate presence and absence (of NOM or biofilm), respectively. For example, Y-NOM N-Biofilm means without biofilm cases in the presence of NOM.
(TIF)

S4 Fig Release of attached *E*. *coli* from biofilters. The release occurs from a) sand and b) biochar-augmented sand columnsdue to a change in background influent condition from synthetic stormwater (ionic strength = 4.7 mM) to DI water (ionic strength = 0 mM). Percent released was calculated by numerical integration of a release curve, which was then normalized by total number of deposited bacteria. Error bars represent standard deviation between replicate measurements (n = 3).
(TIF)

S5 Fig Theoretical DLVO interaction of *E*. *coli* with collectors. Interaction energies a) between sand and *E*. *coli*, and b) between biochar and *E*. *coli* are expressed in terms of KT assuming upper range of the negative surface potential for sand and biochar particles: -30 and -50 mV, respectively. The same Hamaker constant (6.6x10^-21^ J) was assumed for both particles.
(TIF)

S1 Method DLVO modeling details. DLVO forces were calculated using adapted Wieseand-Healy expression for a sphere–flat plate system.
(PDF)
