# Supplementary figures and images for: Escherichia coli Removal in Biochar-Modified Biofilters: Effects of Biofilm
Source: PLoS One. 2016 Dec 1;11(12):e0167489. doi: 10.1371/journal.pone.0167489 (PMC5132165; doi:10.1371/journal.pone.0167489)

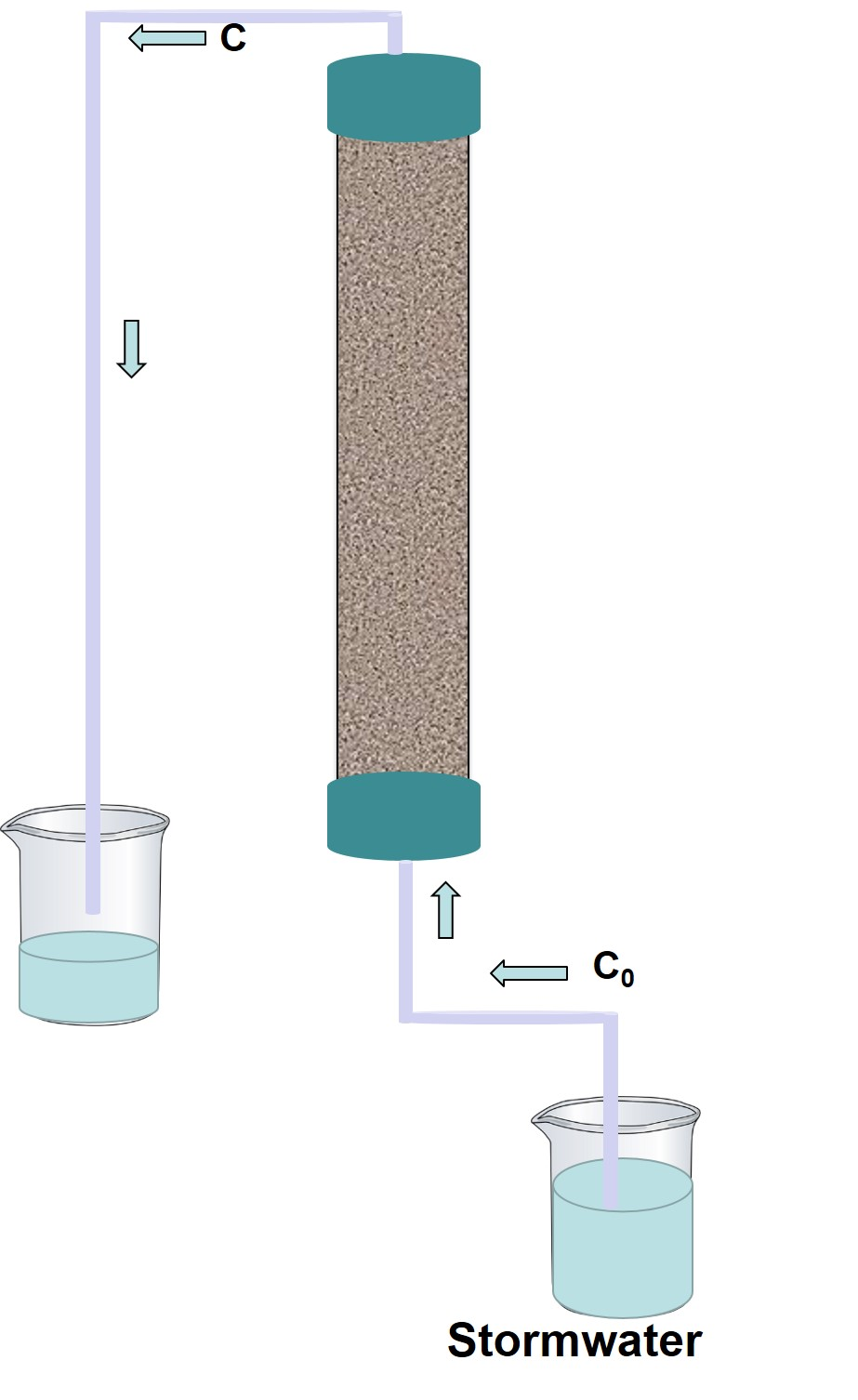

Supplement: S1 Fig — Column experiments were performed at the room-temperature using upflow configuration. (TIF) [file pone.0167489.s001.tif]

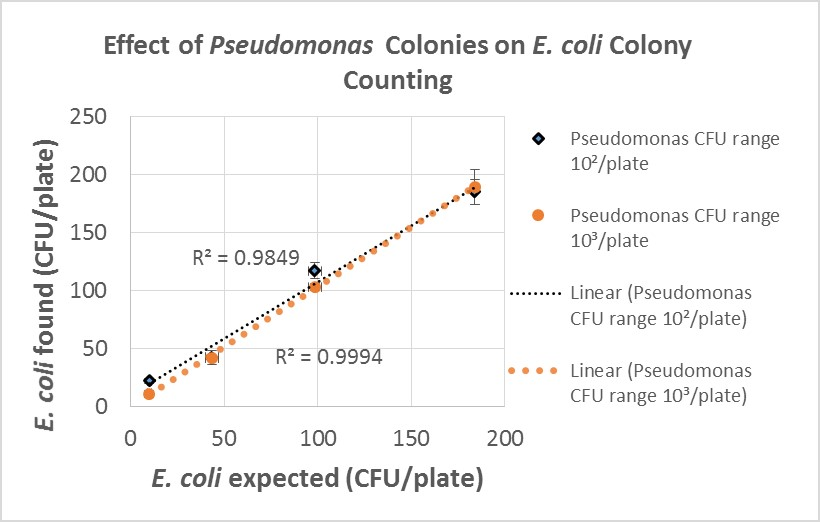

Supplement: S2 Fig — The figure compares the E. coli colony counts between the plates that were spread by E. coli-P.aeruginosa mixture and the plates where a pure E. coli culture were spread. Expected values indicate E. coli concentrations (CFU/plate) observed when plated in the absence of P. aeruginosa. Number of colonies observed in the mixed culture plates are shown as E. coli found (CFU/plate). Error bar represents 1 standard deviation between duplicates (n = 2). (TIF) [file pone.0167489.s002.tif]

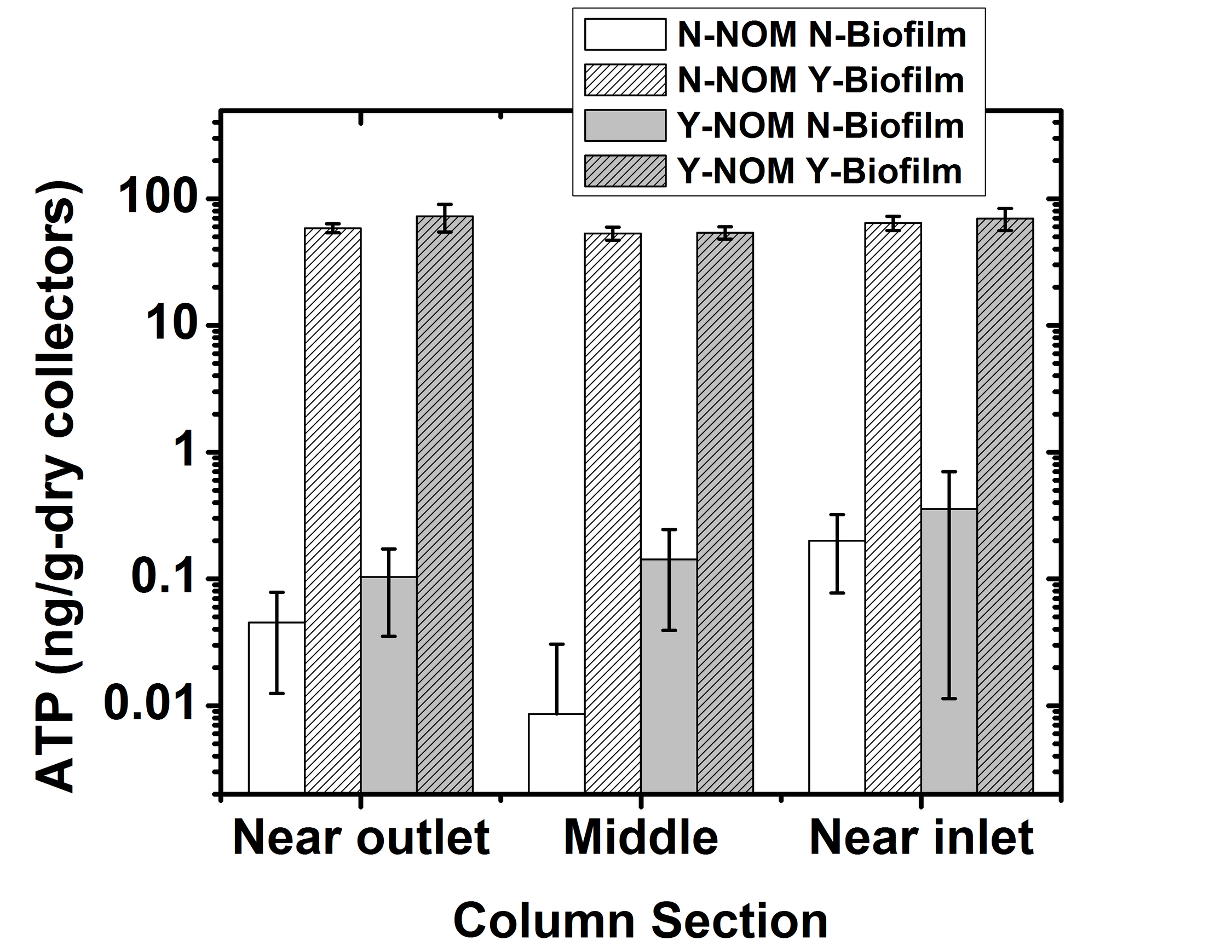

Supplement: S3 Fig — Significantly (p<0.05) higher ATP density (ng/g-dry collectors) was observed in biofilm-coated columns compared to columns without biofilm Error bars represent standard deviation between replicate measurements (n = 3). Y- and N- prefix indicate presence and absence (of NOM or biofilm), respectively. For example, Y-NOM N-Biofilm means without biofilm cases in the presence of NOM. (TIF) [file pone.0167489.s003.tif]

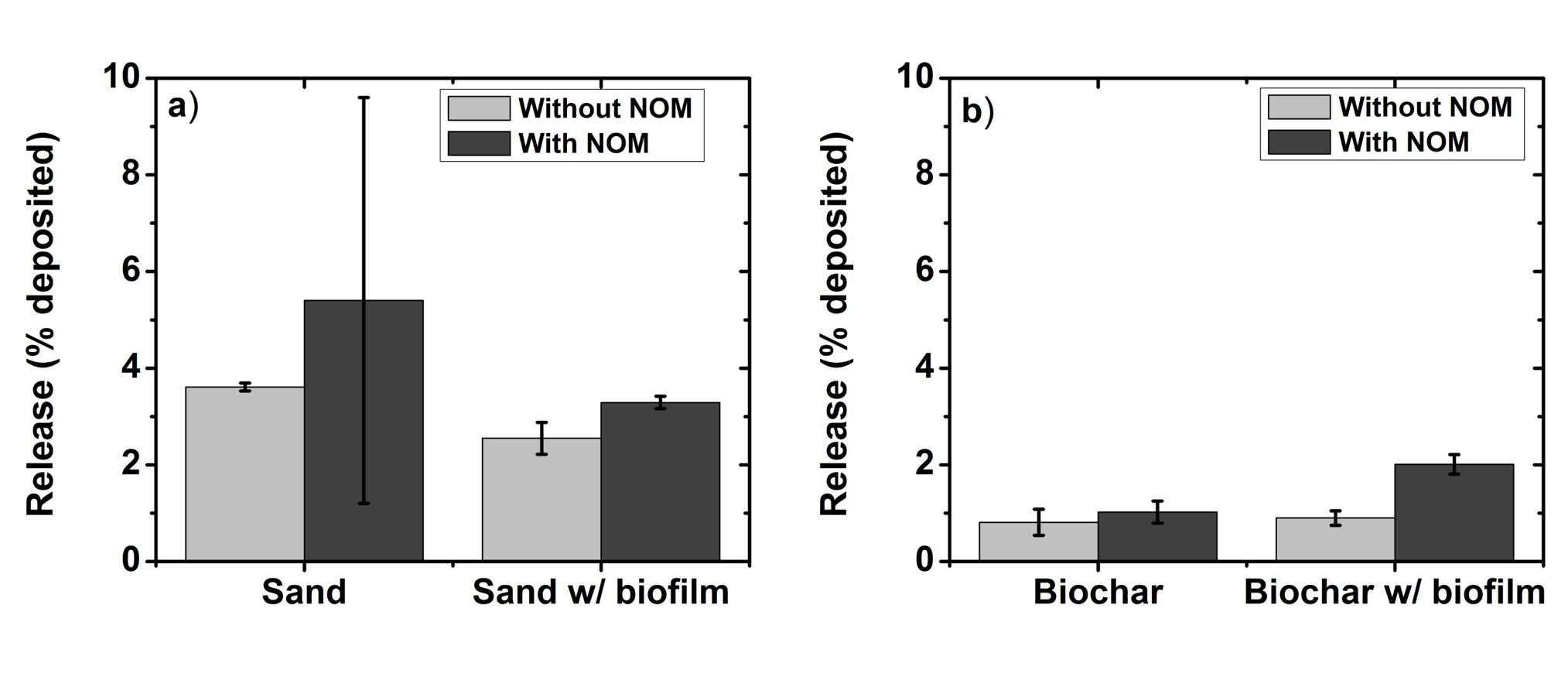

Supplement: S4 Fig — The release occurs from a) sand and b) biochar-augmented sand columnsdue to a change in background influent condition from synthetic stormwater (ionic strength = 4.7 mM) to DI water (ionic strength = 0 mM). Percent released was calculated by numerical integration of a release curve, which was then normalized by total number of deposited bacteria. Error bars represent standard deviation between replicate measurements (n = 3). (TIF) [file pone.0167489.s004.tif]

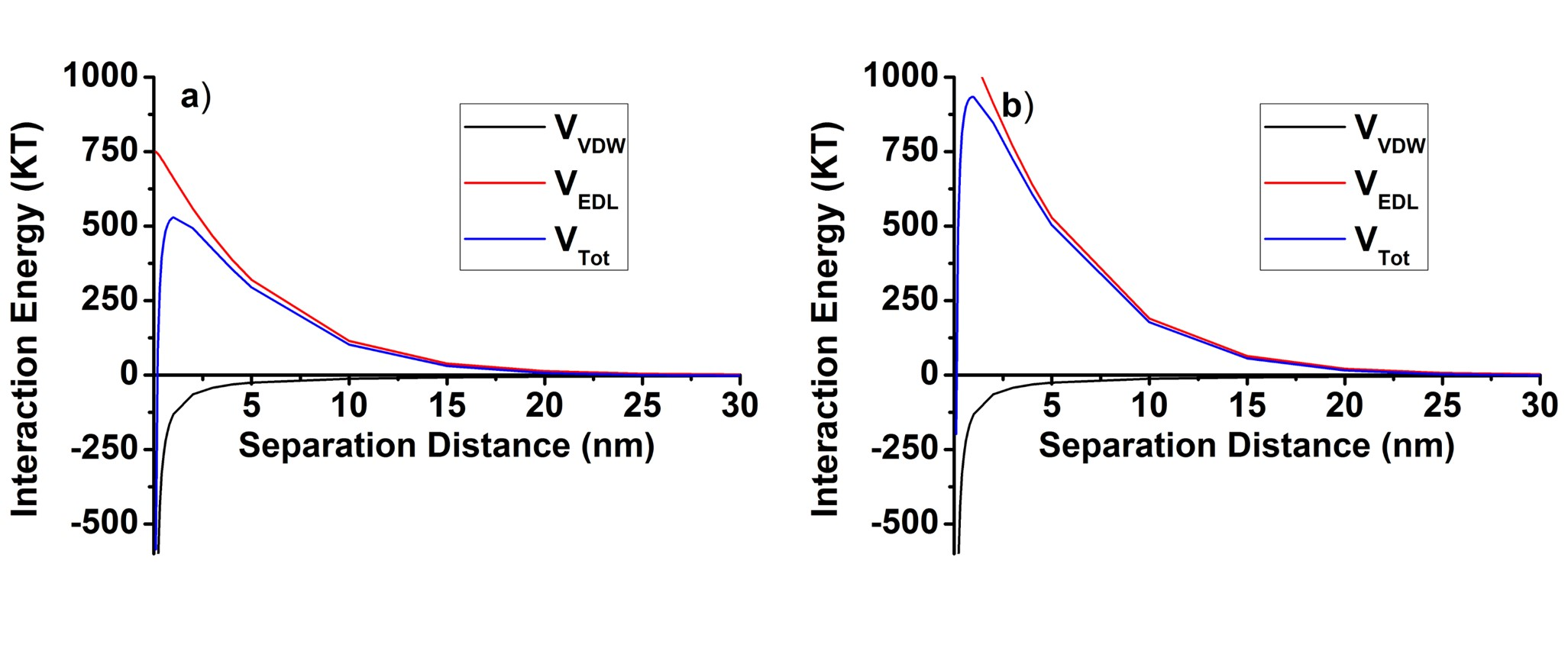

Supplement: S5 Fig — Interaction energies a) between sand and E. coli, and b) between biochar and E. coli are expressed in terms of KT assuming upper range of the negative surface potential for sand and biochar particles: -30 and -50 mV, respectively. The same Hamaker constant (6.6x10^-21^ J) was assumed for both particles. (TIF) [file pone.0167489.s005.tif]
