# Supplementary material for: Escherichia coli Removal in Biochar-Modified Biofilters: Effects of Biofilm
Source: PLoS One. 2016 Dec 1;11(12):e0167489. doi: 10.1371/journal.pone.0167489 (PMC5132165; doi:10.1371/journal.pone.0167489)
Supplement: S1 Method — DLVO forces were calculated using adapted Wieseand-Healy expression for a sphere–flat plate system. (PDF) [file pone.0167489.s006.pdf]

**S1 Method: DLVO modeling details.** DLVO forces were calculated using adapted Wieseand-Healy expression for a sphere–flat plate system [1, 2]. Following equations are used to make the computation:

$$V_{EDL} = 2\pi a n_{\infty} KT \frac{{\Phi_p}^2+{\Phi_e}^2}{2} [\left(\frac{2\Phi_p\Phi_c}{{\Phi_p}^2+{\Phi_c}^2} * \ln\frac{1+\exp(-\kappa h)}{1-\exp(-\kappa h)}\right) + \ln(1-\exp(-2\kappa h))]$$

$$\Phi_i = \frac{Ze\Psi_i}{4KT}$$

$$\kappa = (\frac{e^2 \sum n_{i,\infty} {z_i}^2}{\epsilon\epsilon_0 KT})^{0.5}$$

$$V_{VdW} = -\frac{A_{123}}{6}[\frac{a}{h} + \frac{a}{h+2a} + \ln(\frac{h}{h+2a})]$$

$$V_{Tot} = V_{EDL} + V_{VdW}$$

Where,

$V_{EDL}$= Electrical double layer repulsive energy

$V_{VdW}$= Van-der-Waals attractive energy

$V_{Tot}$= DLVO energy barrier

$a$= Radii of *E. coli* cells [assumed to be 0.5 µm [3]

e= Negative charge of an electron = $1.6*10^{-19}$ C

$n_{i,\infty}$= Equivalent concentration of electrolyte i

$z_i$= Valence of electrolyte i

$n_{\infty}$= Bulk electrolyte density of the stormwater= 4.7 mM equivalent (using the recipe of the synthetic stormwater)

Z = 1 (using the recipe of the synthetic stormwater for a 4.7 mM ionic strength)

$K$= Boltzmann constant = $1.38 \times 10^{-23}$ $m^2$ kg $s^{-2}$ $K^{-1}$

$T$= Temperature= 298 K

$\Psi_p$=Zeta potential of porous media particles =20-50 mV [4, 5]

$\Psi_c$= Zeta potential of *E. coli* cells =36 mV [2]

$\epsilon_0$ = Permittivity of vacuum = 8.854*$10^{-12}$ F/m

$\epsilon$=Relative permittivity of water = 80

h = Separation distance = 1-30 nm (dependent variable)

$A_{123}$= Hamaker constant=6.6x$10^{-21}$ J

References

1. Lin S, Wiesner MR. Exact Analytical Expressions for the Potential of Electrical Double Layer Interactions for a Sphere−Plate System. Langmuir. 2010;26(22):16638-41. doi: 10.1021/la103046w.
2. Truesdail SE, Lukasik J, Farrah SR, Shah DO, Dickinson RB. Analysis of Bacterial Deposition on Metal (Hydr)oxide-Coated Sand Filter Media. Journal of colloid and interface science. 1998;203(2):369-78. Epub 1998/12/16. doi: 10.1006/jcis.1998.5541. PubMed PMID: 9705775.
3. Reshes G, Vanounou S, Fishov I, Feingold M. Cell shape dynamics in Escherichia coli. Biophysical journal. 2008;94(1):251-64.
4. Abit SM, Bolster CH, Cai P, Walker SL. Influence of feedstock and pyrolysis temperature of biochar amendments on transport of Escherichia coli in saturated and unsaturated soil. Environmental science & technology. 2012;46(15):8097-105.
5. Elimelech M, Nagai M, Ko C-H, Ryan JN. Relative Insignificance of Mineral Grain Zeta Potential to Colloid Transport in Geochemically Heterogeneous Porous Media. Environmental Science & Technology. 2000;34(11):2143-8. doi: 10.1021/es9910309.
